# Supplementary material for: A transient amphipathic helix in the prodomain of PCSK9 facilitates binding to low-density lipoprotein particles
Source: J Biol Chem. 2020 Jan 16;295(8):2285–98. doi: 10.1074/jbc.RA119.010221 (PMC7039556; doi:10.1074/jbc.RA119.010221)
Supplement: Supporting Information [file supp_295_8_2285__index.html]

A transient amphipathic helix in the prodomain of PCSK9 facilitates binding to low-density lipoprotein particles — PCSK9 mutations in hypercholesterolemia — A transient amphipathic helix in the prodomain of PCSK9 facilitates binding to low-density lipoprotein particles — PCSK9 mutations in hypercholesterolemia — Supporting Information 

# A transient amphipathic helix in the prodomain of PCSK9 facilitates binding to low-density lipoprotein particles

## Supporting Information

- Supporting Information (to be published online) - Multiple species sequence alignment and supporting experimental data
- Figure S1 - Figure Suppl 1
- Figure S2 - Suppl Figure 2
