# Supplementary material for: A transient amphipathic helix in the prodomain of PCSK9 facilitates binding to low-density lipoprotein particles
Source: J Biol Chem. 2020 Jan 16;295(8):2285–98. doi: 10.1074/jbc.RA119.010221 (PMC7039556; doi:10.1074/jbc.RA119.010221)
Supplement: Supporting Information [file supp_RA119.010221_154343_2_supp_459054_q45vbg.pdf]

Supplemental data

FIGURE LEGENDS

Supplemental Figure S1. A multiple sequence alignment of the human PCSK9 aa 31-52 and other vertebrate species highlights evolutionarily conserved regions containing a preponderance of acidic (shaded red) and hydrophobic (shaded green) residues. The RasMol amino color scheme colors amino acids according to traditional amino acid properties (http://life.nthu.edu.tw/~fmhsu/rasframe/COLORS.HTM). Alignment performed using CLC Sequence Viewer v8.0 software (Qiagen Bioinformatics).

Supplemental Figure S2. R46L mutation in PCSK9 does not affect LDL binding affinity. (**A**) In vitro competition binding of WT and R46L forms of PCSK9 to LDL. LDL particles were incubated with Dylight800-labeled PCSK9 in the presence of increasing concentrations of unlabeled competitor proteins. Reaction mixtures were separated on agarose gels (*top*) and fluorophore-labeled PCSK9 binding to LDL was quantified and fitted to competition binding curves using non-linear regression (*bottom*). (**B**) Inhibitor constants (Ki) obtained from curves in *A*. Error bars represent SEM (n=3).

Supplemental Figure S1

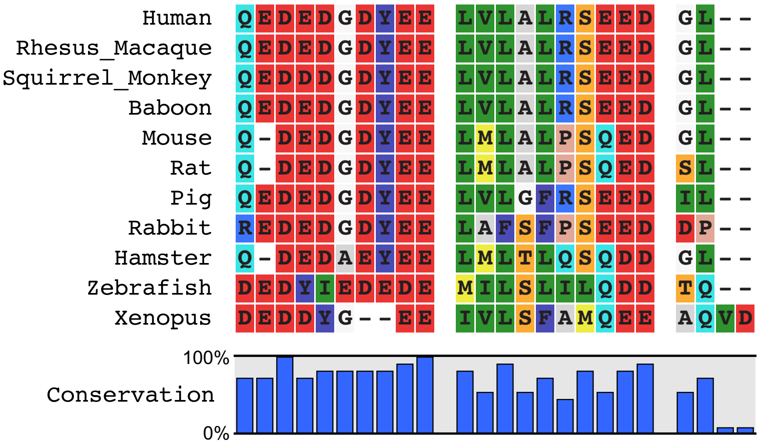

Supplemental Figure S2

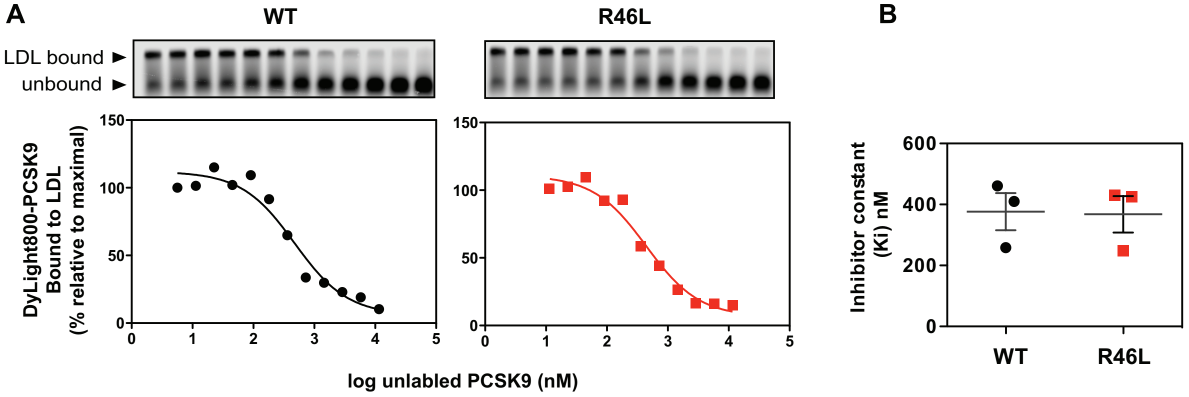

Table 1: Sequences of forward (F) and reverse (R) primers used to mutate C-terminally FLAG-tagged wild-type human PCSK9. Bold, underlined letters indicate mutated base pairs, **|** indicates deletion site.

**Mutant**		**Primer Sequence (5’→ 3’)**
**Δ33-40**	F:	GCGGGCGCCCGTGCGCAGGAG|CTGGTGCTAGCCTTGCGT
	R:	ACGCAAGGCTAGCACCAG|CTCCTGCGCACGGGCGCCCGC
**Gly-Ser 41-46**	F:	GGACGAGGACGGCGACTACGAGGAG**GGT**G**G**G**AGT**G**G**C**GG**G**A**GTT CCGAGGAGGACGGCCTGGCC
	R:	CTCCTCGTAGTCGCCGTCCTCGTCCTCCTGCGCACGGGCGCCCGCG
**A44P**	F:	GGAGCTGGTGCTA**C**CCTTGCGTTCCGAGG
	R:	CCTCGGAACGCAAGG**G**TAGCACCAGCTCC
**L41P**	F:	ACTACGAGGAGC**C**GGTGCTAGCCTTG
	R:	CAAGGCTAGCACC**G**GCTCCTCGTAGT
**R46L**	F:	GGTGCTAGCCTTGC**T**TTCCGAGGAGGACGG
	R:	CCGTCCTCCTCGGAA**A**GCAAGGCTAGCACC
**R496W**	F:	GAGTGGGAAGCGG**T**GGGGCGAGCGCATG
	R:	CATGCGCTCGCCCC**A**CCGCTTCCCACTC
**R469W**	F:	CTCGGGGCCTACA**T**GGATGGCCACAGCCATC
	R:	GATGGCTGTGGCCATCC**A**TGTAGGCCCCGAG
**F515L**	F:	CCACAACGCTTT**A**GGGGGTGAGGGTG
	R:	CACCCTCACCCCC**T**AAAGCGTTGTGG
**Y38F**	F:	CGAGGACGGCGACT**T**CGAGGAGCTGGTGC
	R:	GCACCAGCTCCTCG**A**AGTCGCCGTCCTCG
**Y38L**	F:	CGAGGACGGCGACT**TG**GAGGAGCTGGTGC
	R:	GCACCAGCTCCTC**CA**AGTCGCCGTCCTCG
**Y38A**	F:	GAGGACGGCGAC**GC**CGAGGAGCTGGTG
	R:	CACCAGCTCCTCG**GC**GTCGCCGTCCTC
**Y38E**	F:	CGAGGACGGCGAC**G**A**G**GAGGAGCTGGTGC
	R:	GCACCAGCTCCTC**C**T**C**GTCGCCGTCCTCG
**Y38K**	F:	GAGGACGGCGAC**A**A**A**GAGGAGCTGGTGCTAG
	R:	CTAGCACCAGCTCCTC**T**T**T**GTCGCCGTCCTC
**Y38R**	F:	GAGGACGGCGAC**AGA**GAGGAGCTGGTGCTAG
	R:	CTAGCACCAGCTCCTC**TCT**GTCGCCGTCCTC
