# Supplementary figures and images for: A transient amphipathic helix in the prodomain of PCSK9 facilitates binding to low-density lipoprotein particles
Source: J Biol Chem. 2020 Jan 16;295(8):2285–98. doi: 10.1074/jbc.RA119.010221 (PMC7039556; doi:10.1074/jbc.RA119.010221)

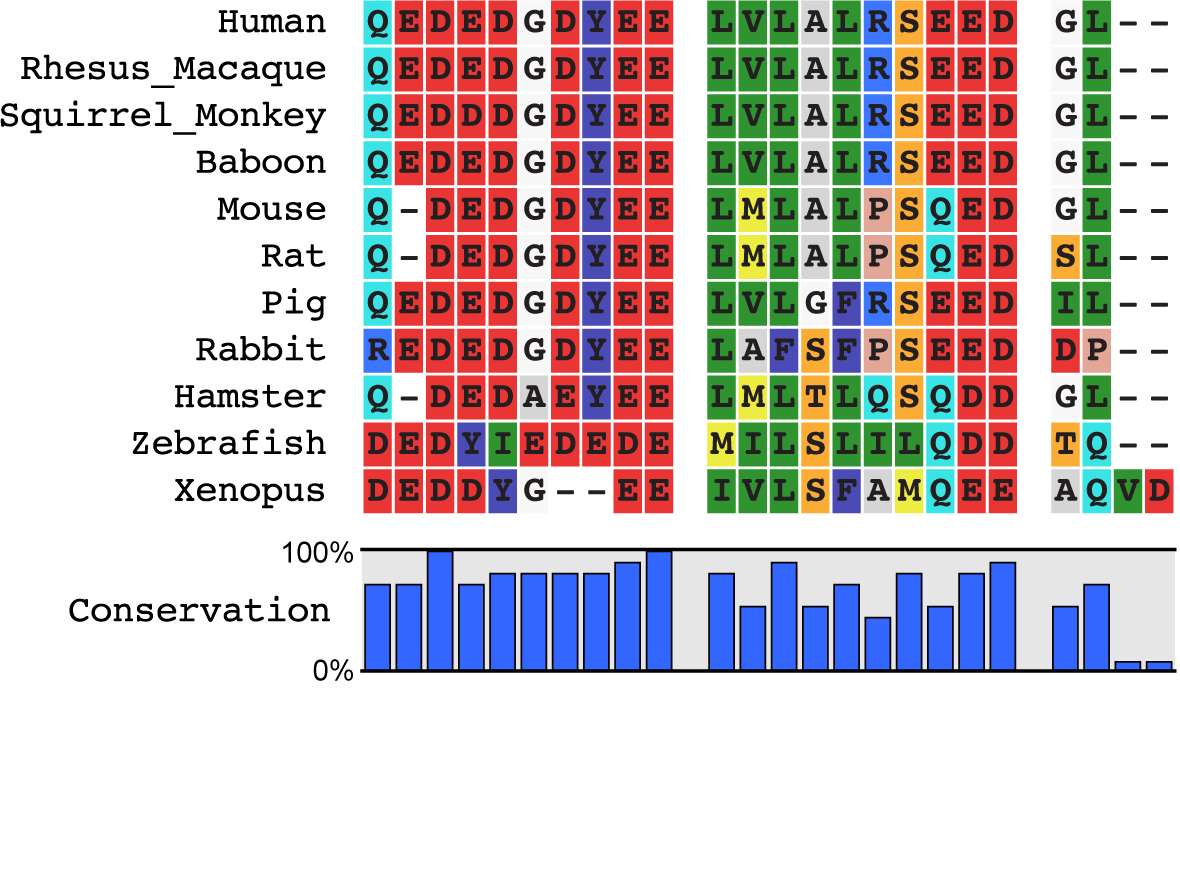

Supplement: Supporting Information [file supp_RA119.010221_154343_2_supp_457423_q47kbs.tif]

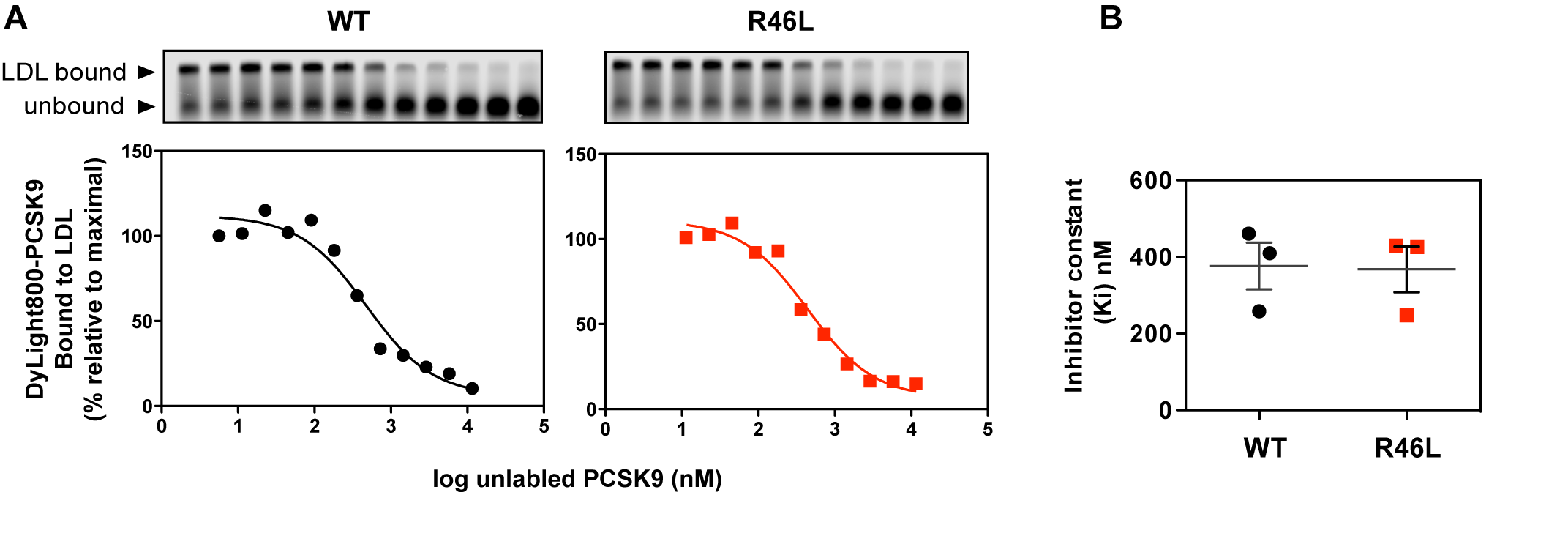

Supplement: Supporting Information [file supp_RA119.010221_154343_2_supp_457424_q47kbs.tif]
